# Low Fibrinogen Levels Are Associated with an Increased Risk of Parenchymal Hematoma in Ischemic Stroke Treated with Intravenous Thrombolysis

**DOI:** 10.3390/jcm15051691

**Published:** 2026-02-24

**Authors:** Libor Šimůnek, Veronika Kunešová, Lucie Burešová, Viktor Weiss, René Jura, Petr Geier, Petra Reková, Daniel Václavík, Martin Šrámek, Robert Mikulík, Roman Herzig

**Affiliations:** 1 Department of Neurology, Comprehensive Stroke Center, University Hospital Hradec Králové, 500 05 Hradec Králové, Czech Republic; libor.simunek@fnhk.cz; 2 Department of Neurology, Faculty of Medicine in Hradec Králové, Charles University, 500 03 Hradec Králové, Czech Republic; 3 Center of Excellence CREATIC, Faculty of Medicine, Masaryk University, 625 00 Brno, Czech Republic; 4 Department of Biostatistics, St. Anne’s University Hospital in Brno, 602 00 Brno, Czech Republic; 5 Department of Public Health, Faculty of Medicine, Masaryk University, 625 00 Brno, Czech Republic; 6 The First Department of Neurology, Comprehensive Stroke Center, St. Anne’s University Hospital in Brno, 602 00 Brno, Czech Republic; 7 The First Department of Neurology, Faculty of Medicine, Masaryk University, 625 00 Brno, Czech Republic; 8 Department of Neurology, Comprehensive Stroke Center, University Hospital Brno, 625 00 Brno, Czech Republic; 9 Department of Neurology, Faculty of Medicine, Masaryk University, 625 00 Brno, Czech Republic; 10 Stroke Center, Department of Neurology, Pardubice Hospital, 532 03 Pardubice, Czech Republic; 11 Department of Neurology, Faculty of Health Studies, University of Pardubice, 532 10 Pardubice, Czech Republic; 12 Department of Neurology, First Faculty of Medicine, Charles University, 121 08 Prague, Czech Republic; 13 Department of Neurology and Center of Clinical Neuroscience, General University Hospital in Prague, 128 08 Prague, Czech Republic; 14 Stroke Center, Department of Neurology, AGEL Hospital Ostrava–Vítkovice, 703 00 Ostrava–Vítkovice, Czech Republic; 15 AGEL Educational and Research Institute, 796 04 Prostějov, Czech Republic; 16 Department of Neurology, Comprehensive Stroke Center, Military University Hospital Prague, 169 02 Prague, Czech Republic; 17 Cerebrovascular Research Program, International Clinical Research Centre, St. Anne’s University Hospital in Brno, 602 00 Brno, Czech Republic; 18 Department of Neurology, Comprehensive Stroke Center, T. Baťa Regional Hospital, 762 75 Zlín, Czech Republic

**Keywords:** ischemic stroke, intravenous thrombolysis, alteplase, fibrinogen, intracerebral hemorrhage, parenchymal hematoma

## Abstract

**Background**: Intravenous thrombolysis (IVT), used in acute ischemic stroke (AIS), may be complicated by the development of intracranial hemorrhage. The role of fibrinogen levels, including their decrease, as a possible predictor of intracranial hemorrhage, has not yet been fully clarified. We aimed to evaluate the association between fibrinogen levels and their decrease 6 and 24 h after IVT and the risk of parenchymal hematoma (PH), as the clinically most significant type of intracranial hemorrhage. **Methods**: In an observational, nationwide, multicenter study, data from adult patients who underwent IVT for AIS from the Registry of Stroke Care Quality (RES-Q) in the Czech Republic (2019–2021) were analyzed. An association between fibrinogen levels and their decrease 6 and 24 h after IVT and the risk of PH was assessed. **Results**: We analyzed a set of 27 patients with PH (13 males; median age 78.0 years) and a control group (CG) of 97 patients without intracranial hemorrhage (58 males; median age 78.0 years). Fibrinogen levels 6 h after IVT (median 1.93 [PH] vs. 2.57 [CG] g/L, *p* = 0.012) and the ratio of baseline fibrinogen to fibrinogen 6 h after IVT (median 1.78 [PH] vs. 1.26 [CG]; *p* = 0.008) were associated with the development of PH. The optimal cut-off value of fibrinogen 6 h after IVT for predicting PH was <2.0 g/L. **Conclusions**: Fibrinogen levels 6 h after IVT and the ratio of baseline fibrinogen to fibrinogen 6 h after IVT are associated with an increased risk of PH in patients with acute ischemic stroke treated with IVT.

## 1. Introduction

Intravenous thrombolysis (IVT) using recombinant tissue plasminogen activator (rtPA; alteplase) is an established and recommended recanalization therapy for acute ischemic stroke. The efficacy of IVT is well documented and has clear benefits for patients in terms of functional independence, as measured by the modified Rankin Scale (mRS). The number needed to treat (NNT) to achieve an mRS 0–1 varies depending on the therapeutic window: for 0–3 h, the NNT is reported to be between 8–10, while for 3–4.5 h it ranges between 12–19 [1,2]. Therefore, the primary goal is to administer IVT as quickly as possible to maximize the likelihood of recanalization and minimize the risks of intracranial hemorrhage, permanent neurological deficits, and death. Studies show that every 15 min of delay in administering IVT reduces the likelihood of achieving an mRS 0–1 by 16% [3]. Despite its benefits, one of the most serious complications of IVT is intracranial hemorrhage, occurring in approximately 6–7% of cases according to the classification of the National Institute of Neurological Disorders and Stroke (NINDS) [4]. Current evidence supports the efficacy of IVT within 4.5 h of symptom onset; however, recent studies suggest potential benefits even within the 4.5–9 h time window or in cases where the time of onset is unknown, provided that patients are selected based on multimodal computed tomography (CT) imaging [5]. However, administration of alteplase within this extended time window is associated with an increased risk of intracranial hemorrhage within 48 h, which can significantly affect patient outcomes [3].

Intracranial hemorrhage after IVT can be divided into intracerebral hemorrhage (ICH) and other intracranial hemorrhage (e.g., subarachnoid, intraventricular, or subdural hemorrhage). The European Cooperative Acute Stroke Study (ECASS) classification further divides ICH into hemorrhagic infarction (HI) and parenchymal hematoma (PH) [6], and further subdivides them into subtypes (HI1, HI2, PH1, PH2). Several meta-analyses show that type 2 PH (hematoma affecting > 30% of the ischemic area with significant mass effect) occurs in 6.8% of patients treated with IVT compared to 1.3% in control groups, representing a six-fold increase in risk (odds ratio [OR] = 5.55; 95% confidence interval [CI]: 4.01–7.70) [7]. However, in addition to ICH within the area of ischemia, IVT may also be complicated by the occurrence of so-called remote hematoma, e.g., ICH outside the area of ischemia or subarachnoid (SAH) or intraventricular hemorrhage (IVH). These remote hematomas may be clinically significant, but in some cases may not have a direct clinical impact.

Several risk factors for ICH after IVT have been identified, including advanced age, arterial hypertension, heart disease, hyperglycemia, extreme body weight, previous stroke, delayed thrombolysis, and higher doses of alteplase [8,9]. Several studies have shown that a significant reduction in fibrinogen levels correlates with an increased risk of ICH [10,11,12,13,14,15]. The difference in recommendations regarding when to measure fibrinogen levels after IVT suggests that there is no consensus on optimal monitoring. Some guidelines recommend measurement at 6 and 24 h after IVT [5], while others do not specify routine testing. Given that fibrinogen is one of the few potentially modifiable risk factors for ICH, optimizing its monitoring and possible substitution may represent a means of mitigating hemorrhagic complications after IVT. Currently, the guidelines of the Czech Stroke Society of the Czech Neurological Society recommend testing coagulation parameters, including fibrinogen levels, before IVT and 6 and 24 h after IVT, along with a follow-up CT scan after 22–36 h, unless clinical conditions require imaging earlier [5]. However, more data are needed to determine whether fibrinogen monitoring at specific time intervals can be used as a predictor of ICH risk.

This study aimed to evaluate the association between fibrinogen levels and their decrease after IVT and the risk of ICH. Specifically, we investigated whether baseline fibrinogen levels and their decrease 6 and 24 h after IVT correlated with an increased incidence of PH, as the clinically most significant type of ICH.

## 2. Materials and Methods

### 2.1. Study Design and Setting

This retrospective nationwide multicenter observational study was conducted in seven hospitals in the Czech Republic. Data were analyzed from adult patients who underwent IVT alone for acute ischemic stroke with a standard dose of 0.9 mg/kg of rtPA (Actilyse^®^; Boehringer Ingelheim, Ingelheim am Rhein, Germany) following the national guidelines [5,16], prospectively collected in the RES-Q (Registry of Stroke Care Quality) registry in the Czech Republic between 2019 and 2021. RES-Q is a prospective, multicenter international data collection platform developed under the auspices of the European Stroke Organisation and currently used worldwide (in the Czech Republic since 2019) to help physicians monitor and improve the quality of care for stroke patients, including confirming the safety and efficacy of IVT in clinical practice [17]. According to a uniform protocol, clinical and demographic data on patients treated with IVT in the Czech Republic are stored in this registry.

### 2.2. Study Population and Observed Parameters

This retrospective study included adult patients aged 18 to 90 years who had a neurological deficit of ≥2 points on the National Institutes of Health Stroke Scale (NIHSS) at admission. Patients who underwent concomitant endovascular or surgical treatment for stroke or fibrinogen replacement between IVT and follow-up CT scan were excluded from the study. All patients underwent CT scanning 22–36 h after initiation of IVT to detect possible intracranial hemorrhagic complications.

Patients with any intracranial hemorrhage were included in the observation group. A control group, consisting of acute ischemic stroke patients without intracranial hemorrhagic complications, was created using a 1:1 approach, i.e., each patient with intracranial hemorrhagic complications after IVT was randomly assigned to a patient without such complications with a corresponding NIHSS score, age, and sex. Other parameters monitored included the presence of vascular risk factors (arterial hypertension, diabetes mellitus [DM]—all individuals diagnosed with DM or taking antidiabetic drugs were classified as diabetics), pharmacological history (antihypertensive drugs, antidiabetic drugs, acetylsalicylic acid, clopidogrel, warfarin, dabigatran, rivaroxaban, apixaban, edoxaban, unfractionated heparin, low molecular weight heparin [LMWH] in prophylactic dose, LMWH in therapeutic dose), laboratory parameters (baseline fibrinogen level, fibrinogen after 6 h and after 24 h, international normalized ratio [INR], activated partial thromboplastin time [aPTT], platelet count, urea, creatinine, estimated glomerular filtration rate), clinical parameters (baseline systolic and diastolic blood pressure, NIHSS score), and initial CT scan results (Alberta Stroke Program Early CT Score [ASPECTS] and the presence of intracranial hemorrhage) (Table 1).

Δ fibrinogen was defined as the initial fibrinogen value minus the value after 6 h or after 24 h, respectively; a higher positive value, therefore, indicates a greater decrease. The fibrinogen ratio was calculated as the initial fibrinogen value/value after 6 h (or after 24 h, respectively). A higher positive value, therefore, indicates a multiple decrease in fibrinogen levels.

### 2.3. Classification of Intracranial Hemorrhage

Based on the results of the follow-up CT scan, intracranial hemorrhage was classified according to ECASS criteria as HI1, HI2, PH1, PH2, and intracranial hemorrhage outside the ischemic area (i.e., remote ICH, SAH, or IVH). HI1 was defined as small petechiae along the margins of the infarct; HI2, as confluent petechiae within the infarcted area but without space-occupying effect; PH1, as blood clots in ≤30% of the infarcted area with some slight space-occupying effect; and PH2, as blood clots in >30% of the infarcted area with a substantial space-occupying effect [6].

### 2.4. Statistical Analysis

A comparison of categorical variables between groups (PH vs. no PH) was performed using the chi-square test. Since most continuous variables did not meet the assumption of normal distribution, differences between groups were assessed using the non-parametric Mann–Whitney U test.

The optimal cut-off value of fibrinogen concentration for predicting the development of PH was determined using receiver operating characteristic (ROC) curve analysis with the Youden index to identify the point of maximal discrimination. For each parameter evaluated, the areas under the ROC curve (AUC), sensitivity, and specificity were calculated. Since the risk of hemorrhage increases with decreasing fibrinogen levels, several variables were transformed (including sign inversion) to ensure the correct direction of association and accurate estimation of the optimal cut-off value.

A logistic regression model was used to assess the combined effect of clinical and laboratory factors on the risk of PH. The model included fibrinogen levels 6 h after IVT (g/L) and the presence of DM, as the two groups differed significantly in the proportion of diabetic patients. The model provided OR with 95% CI, expressing the multiplicative change in the odds of developing a hemorrhagic complication in relation to these predictors.

The following continuous variables were also analyzed: Δ fibrinogen (difference between baseline and 6-h level) and fibrinogen ratio (baseline/6-h value). Both variables were assessed using nonparametric tests and included in logistic regression models to explore their potential association with the occurrence of PH. A two-tailed *p*-value < 0.05 was considered statistically significant.

All statistical analyses were performed using R software, version 4.2.1 (R Foundation for Statistical Computing, Vienna, Austria).

### 2.5. Definition of Variables

- DM: patients diagnosed with DM or taking antidiabetic drugs.
- Δ fibrinogen: defined as the difference between baseline and 6-h levels; higher positive values indicate a greater decrease.
- Fibrinogen ratio: calculated as baseline/6-h value; higher values correspond to a more significant relative decrease in fibrinogen concentration.
- Baseline fibrinogen: measured immediately before the start of IVT.
- Fibrinogen at 6 h and 24 h: follow-up samples taken 6 h and 24 h after IVT administration.
- PH: defined according to ECASS criteria [6].
- NIHSS: score at admission, representing the severity of neurological deficit.
- ASPECTS: score evaluated on baseline brain CT.
- Blood pressure: systolic and diastolic blood pressure at admission, considered a continuous variable.
- Other laboratory parameters: INR, aPTT, creatinine, urea, platelet count, and estimated glomerular filtration rate (eGFR) measured using standard laboratory methods.

## 3. Results

The study population included 318 patients: 159 in the observation group (intracranial hemorrhagic complications after IVT) and 159 in the control group (no intracranial hemorrhagic complications after IVT). A 1:1 matching strategy was used (same NIHSS values, age, sex). Treatment was complicated by the development of PH in 53 cases (18 cases of PH1, 35 cases of PH2, of which 16 cases of PH were also associated with hemorrhagic complications outside the area of ischemia)—these hemorrhagic complications are considered clinically significant. The bleeding complication was HI type without other bleeding in 71 cases (55 cases exclusively HI1, 16 cases exclusively HI2)—these bleeding complications are considered clinically insignificant. The remaining 35 cases involved bleeding outside the area of ischemia, of which 4 cases involved a combination with HI, and 31 cases involved exclusively bleeding outside the area of ischemia (Table 2).

**Figure 1:**
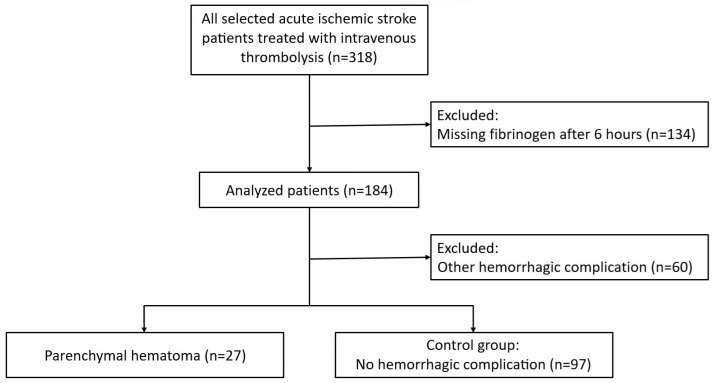
Study flowchart.

We excluded 134 patients from the study population because their fibrinogen values after 6 h were not available. After this exclusion, we were left with a group of 27 patients with PH (PH1 only, PH2 only, PH1 + remote bleeding, PH2 + remote bleeding), a group of 60 patients with other intracranial hemorrhagic complications, and a group of 97 patients without hemorrhagic complications. After excluding 60 patients with hemorrhagic complications other than PH, the final statistical analysis included 124 patients. Accordingly, we compared a group of 27 patients with PH with a control group of 97 patients (Figure 1).

The basic characteristics of the patients are shown in Table 3.

The results of fibrinogen levels (baseline and after 6 and 24 h), Δ fibrinogen, and fibrinogen ratio in individual groups are shown in Table 4.

Given the demonstrated significance of the difference in fibrinogen levels between the two groups 6 h after IVT, we performed the following subanalyses of fibrinogen levels 6 h after IVT.

**Figure 2:**
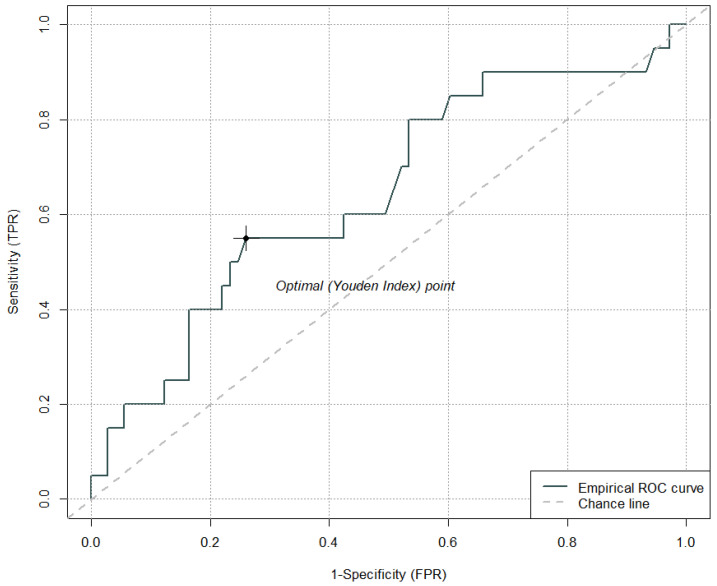
Cut-off value of fibrinogen levels 6 h after intravenous thrombolysis for the development of parenchymal hematoma.

The cut-off value for fibrinogen levels 6 h after IVT that has the optimal sensitivity and specificity ratio for the development of PH was <2.0 g/L (sensitivity 55.0%, specificity 74.0%); AUC = 0.65 (Figure 2). In the ROC curve analysis for fibrinogen levels 6 h after IVT, where lower values were associated with a higher risk of PH, the values were multiplied by −1 to standardize the direction of effects across all parameters. As a result of this transformation, the optimal cut-off is expressed as a negative number. After back-transformation, we report this result as a cut-off value of <2.0 g/L.

Also, according to the results of logistic regression, fibrinogen levels 6 h after IVT are a significant predictor of PH development. An increase in fibrinogen levels of 1 g/L reduces the odds of PH by almost half (Table 5).

**Figure 3:**
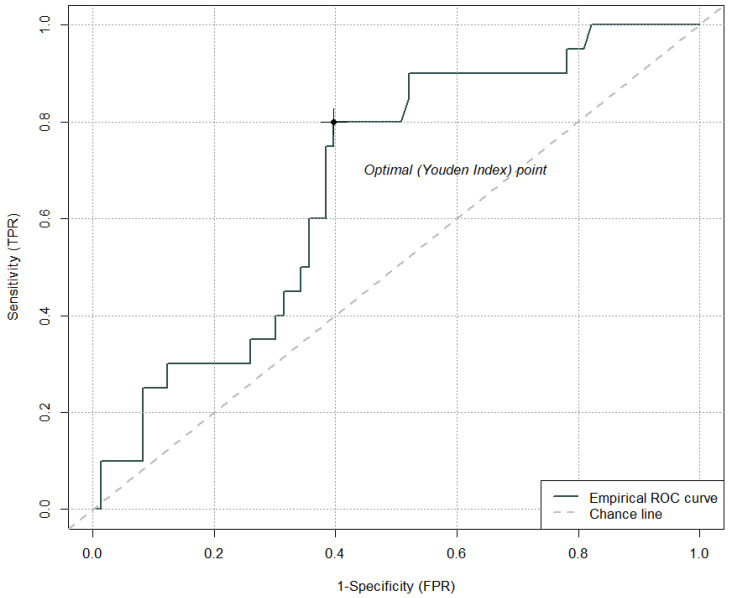
Cut-off value of Δ fibrinogen (baseline vs. 6 h after intravenous thrombolysis) for the development of parenchymal hematoma.

Δ fibrinogen (baseline vs. 6 h after IVT) did not prove to be a significant predictor for the development of PH. The cut-off value of Δ fibrinogen (baseline vs. 6 h after IVT) that has the optimal sensitivity and specificity ratio for the development of PH corresponds to a difference in fibrinogen levels of 0.84 g/L (sensitivity 80.0%, specificity 60.0%); AUC = 0.67 (Figure 3).

According to the results of logistic regression, Δ fibrinogen (baseline value vs. 6 h after IVT) is not a significant predictor for the development of PH (Table 6).

**Figure 4:**
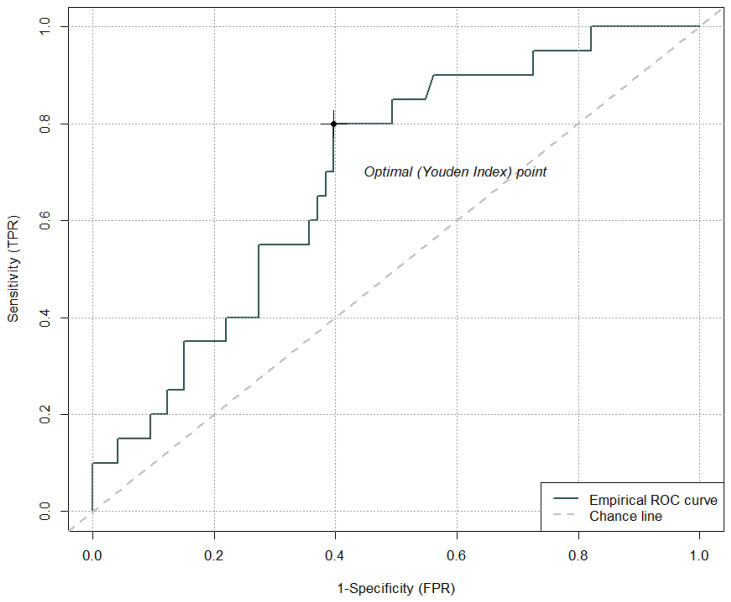
Cut-off value for the fibrinogen ratio (baseline vs. 6 h after intravenous thrombolysis) for the development of parenchymal hematoma.

The cut-off value for the fibrinogen ratio (baseline vs. 6 h after IVT) that has the optimal sensitivity and specificity ratio for the development of PH is 1.34 (sensitivity 80.0%, specificity 60.0%); AUC = 0.69 (Figure 4).

Also, according to the results of logistic regression, the fibrinogen ratio (baseline vs. 6 h after IVT) is a significant predictor for the development of PH. An increase in the fibrinogen ratio (baseline vs. 6 h after IVT) of approximately 2.7× is associated with an approximately twofold increase in the odds of PH (Table 7).

The results of other laboratory parameters are presented in Table 8. INR test results were available for only 13 patients, and aPTT results for only 17 patients; therefore, no statistical comparison of these parameters was performed.

## 4. Discussion

Despite the well-known benefits of IVT in the treatment of acute ischemic stroke, its use is limited by the risk of ICH. Although several predictors of ICH have been identified, the role of fibrinogen depletion as a potentially modifiable risk factor remains poorly understood. Our study aimed to refine knowledge about fibrinogen dynamics after IVT and its role in ICH risk stratification. By identifying the optimal timing and threshold values for fibrinogen monitoring, this research could contribute to improving patient outcomes and optimizing individualized stroke treatment protocols. To the best of our knowledge, our study was the first to evaluate the significance of the decrease in fibrinogen levels specifically at 6 and 24 h after IVT in acute ischemic stroke as a possible predictor of the development of PH, the most clinically significant type of ICH.

In our study, we observed a decrease in fibrinogen levels after administration of alteplase in both patients with PH and patients without intracranial hemorrhagic complications. This decrease and its mechanism have been described in the literature. Alteplase binds to plasminogen within the clot, converting it to plasmin, a proteolytic enzyme capable of breaking cross-links between fibrin molecules and thus dissolving clots. However, the affinity of rtPA is not exclusive to thrombus fibrin. It also binds to circulating fibrinogen, resulting in fibrinogen degradation [18,19]. In clinical practice, the treatment of ischemic stroke with IVT using tenecteplase is becoming increasingly widespread. Compared to alteplase, tenecteplase has a higher affinity for fibrin clots. This may lead to a lower degree of fibrinogen depletion and potentially a lower risk of symptomatic intracranial hemorrhage [20,21].

The initial fibrinogen level was not a predictor of PH development in our cohort. Several previous studies have shown that higher fibrinogen levels before IVT treatment are associated with a statistically significant occurrence of intracerebral hemorrhagic complications [11,12,22,23,24]. The mechanism by which patients with increased fibrinogen are more prone to hemorrhagic transformation is unclear. However, some studies have not confirmed this association [13,25], and in one study, the authors even found that the incidence of hemorrhagic transformation was higher when baseline fibrinogen levels were extremely low (less than 1.5 g/L) [26].

In our study, we demonstrated that patients with PH, the most severe form of hemorrhagic complication, had significantly lower fibrinogen levels 6 h after IVT administration than patients in the control group (1.93 vs. 2.57 g/L; *p* = 0.012). The fact that low fibrinogen levels after IVT are associated with the risk of intracranial hemorrhage has been well documented [10,11,12,13,14,15]. Some of these studies have shown that the difference between baseline and control values correlates better with the occurrence of hemorrhagic complications than the absolute control value of fibrinogen level [12,14]. We have demonstrated that the difference between baseline and control fibrinogen values 6 h after IVT is not a suitable predictor for the development of PH, but rather the ratio of these fibrinogen values.

In our dataset, we divided intracranial hemorrhage into subtypes based on CT scan findings, namely HI1, HI2, PH1, PH2, and remote, which has only been done in one study to date [13]. This study demonstrated an association between hemorrhage and low fibrinogen levels in the PH type, but not in the HI type. Other studies have typically used one of the definitions of symptomatic intracranial hemorrhage based on an assessment of clinical deterioration.

Our study shows that fibrinogen levels 24 h after IVT do not correlate with the occurrence of bleeding complications, i.e., checking levels after 24 h may not provide additional predictive value. Previous studies of patients with myocardial infarction treated with alteplase have shown that fibrinogen reaches its lowest point sometime between 90 min and 3 h [27]. Previous studies in patients with stroke most often evaluated the decrease in fibrinogen levels 2 h after IVT [12,13,15]. Attention should be focused on changes in fibrinogen levels in the early stages after IVT administration (0–6 h), also because the median time from alteplase infusion to confirmation of symptomatic intracranial hemorrhage is 7 h 50 min [28]. Overall, however, it should be emphasized that the timing of fibrinogen sampling relative to the onset of bleeding is uncertain.

Fibrinogen replacement is available in hospitals (Haemocomplettan P, CSL Behring GmbH, Marburg, Germany; or Fibryga, Octapharma [IP] SPRL, Brussels, Belgium). However, there are no official recommendations regarding the decrease in fibrinogen after IVT that would justify fibrinogen replacement, or whether it should be replaced at all. The results of our study, which examined fibrinogen levels 6 h after IVT and showed that the optimal fibrinogen threshold for predicting PH was <2.0 g/L (at this value, the combination of specificity and sensitivity is most satisfactory: sensitivity 55.0%, specificity 74.0%), may serve as a guide. It should be noted that when calculating the cut-off value, the calculated AUC did not exceed 0.7 for any curve. The fibrinogen level 6 h after IVT is therefore not an ideal variable for defining patients at risk of bleeding complications. There will always be a certain number of false positive and false negative results. Therefore, fibrinogen parameters should be interpreted in conjunction with established clinical and radiological risk factors. A similar cut-off value was sought by Vandelli et al. in 2015 [12] and Yan et al. in 2019 [13], in both cases in control fibrinogen samples taken 2 h after IVT. In the first of these studies, the cut-off value was <2.0 g/L for predicting any intracranial hemorrhage, including HI [12]. In the second of these studies, a cut-off value of <2.5 g/L (sensitivity 72.0%, specificity 61.1%) and a cut-off value for fibrinogen depletion of >0.5 g/L (sensitivity 80.0%, specificity 51.0%) were predictive of the development of symptomatic intracranial hemorrhage in patients treated with IVT alone [13]. A cut-off value of <2.0 g/L for control fibrinogen was also used to define fibrinogen depletion after IVT in a recent study by Theodorou et al. [29]. However, our study is the first to confirm the predictive significance of the fibrinogen control cut-off value of <2.0 g/L specifically for the development of PH, the most clinically significant type of ICH. Therefore, a fibrinogen level < 2.0 g/L 6 h after IVT may justify more careful monitoring of neurological deficits and vital signs, earlier repeat imaging, or lower thresholds for intensive care unit treatment and neurosurgeon consultation.

To our knowledge, only one study has evaluated fibrinogen replacement therapy after IVT administration. Vandelli et al. in 2019 [30] analyzed a group of 39 patients who experienced a critical decrease in fibrinogen levels 2 h after the end of IVT (absolute value < 1 g/L in patients without ICH and absolute value < 2 g/L in cases of ICH, or in both cases, a relative decrease in fibrinogen level before and after thrombolysis > 30%). All 39 patients were administered fibrinogen concentrate (Haemocomplettan P). Two patients developed pulmonary embolism, while the other patients had no thrombotic complications. Although this study demonstrated the relative safety of fibrinogen replacement, it did not assess its efficacy. Experience in cardiac surgery has also shown that the administration of fibrinogen concentrate was not associated with an increased risk of mortality and thromboembolic events [31,32].

Our study has several limitations. First, retrospective design carries the risk of selection bias and heterogeneity between centers, particularly regarding the laboratory methods used for fibrinogen measurement and timing of blood sampling. A second limitation relates to incomplete data for some patients and the absence of a uniform definition of clinically significant decreases in fibrinogen levels in the literature. These factors may affect the accuracy and interpretability of the findings to some extent. A third limitation of our study concerns the timing of the follow-up CT scan. In accordance with national guidelines [5], a follow-up CT scan was performed 22–36 h after IVT, so it was uncertain whether intracranial hemorrhage had already occurred before the fibrinogen sample was taken 6 h after IVT or whether it developed later, which limits the interpretation of causal relationships. This uncertainty limits the ability to establish a clear temporal relationship between the decrease in fibrinogen levels and the onset of bleeding. From a clinical perspective, the moment when fibrinogen concentration falls below a critical level is of paramount importance, as is whether its timely replacement can prevent hemorrhagic complications. The correct timing of sampling and possible intervention is therefore crucial for the clinical applicability of these findings. In a study of patients treated with alteplase for myocardial infarction, fibrinogen reached its lowest levels between 90 min and 3 h after IVT [27]. This interval may therefore represent the optimal time window for targeted fibrinogen measurement and for its potential replacement.

Future research should focus on a prospective multicenter study that would confirm or refute the predictive role of fibrinogen levels and their development over time in the context of IVT treatment. Such a study would aim to carefully monitor the dynamics of the decrease in fibrinogen levels and simultaneously perform follow-up CT scans. Such a design would allow for a better understanding of the pathophysiological relationship between the decrease in fibrinogen levels and the development of PH after IVT, while also providing a basis for testing the safety and efficacy of early replacement therapy. At the same time, it would be appropriate to expand the range of monitored hemostatic parameters, for example, to include D-dimer, fibrin degradation products, or plasminogen levels, and to monitor their dynamic changes over time. The data obtained in this manner could contribute to the development of a comprehensive predictive model for the risk of hemorrhagic complications after IVT and, subsequently, to the revision of current clinical recommendations.

## 5. Conclusions

Fibrinogen levels 6 h after IVT were significantly lower in patients with PH than in patients without bleeding complications. The optimal cutoff value for predicting PH was <2.0 g/L. Although the absolute decrease in fibrinogen 6 h after IVT was not a significant predictor of PH, the ratio of baseline fibrinogen to fibrinogen 6 h after IVT showed predictive value. Baseline fibrinogen levels and fibrinogen levels 24 h after IVT were not associated with PH. Further prospective studies are needed to confirm these findings and to determine whether early targeted testing and replacement of fibrinogen can reduce the risk of PH after thrombolytic therapy.

## Figures and Tables

**Table 1:** Observed parameters.

Parameter	Type of Variable	Time of Measurement	Units/Definition
Demographics			
Sex	Categorical		Male/female
Age	Quantitative	At admission	Years
Vascular risk factors			
Arterial hypertension	Dichotomous (yes/no)	At admission	Diagnosis in history or use of chronic antihypertensives (excluding diuretics)
Diabetes mellitus	Dichotomous (yes/no)	At admission	Diagnosis in history or use of chronic antidiabetics
Pharmacological history			
Antiplatelet therapy	Categorical	At admission	Presence of chronic medication: acetylsalicylic acid, clopidogrel
Anticoagulant therapy	Categorical	48 h before IVT	Warfarin, dabigatran, rivaroxaban, apixaban, edoxaban, LMWH—prophylactic dose, LMWH—therapeutic dose, UFH
Laboratory parameters			
Fibrinogen level	Quantitative	0 h, 6 h, 24 h after IVT	g/L
INR	Quantitative	At admission	Normal range: 0.8–1.2
aPTT	Quantitative	At admission	Normal range: 0.8–1.2
Platelets	Quantitative	At admission and the first value after IVT	10^9^/L
Urea	Quantitative	At admission	mmol/L
Creatinine	Quantitative	At admission	μmol/L
eGFR	Quantitative	At admission	mL/s
Clinical parameters			
Systolic blood pressure	Quantitative	At admission	mmHg
Diastolic blood pressure	Quantitative	At admission	mmHg
NIHSS	Ordinal	At admission	Score 0–35
Initial CT scan results			
ASPECTS	Ordinal	Before IVT	Score 0–10
Presence of intracranial hemorrhage	Categorical	Within 36 h post-IVT	HI1, HI2, PH1, PH2, SAH, IVH, SDH, remote

aPTT—activated partial thromboplastin time; ASPECTS—Alberta Stroke Program Early CT Score; CT—computed tomography; eGFR—estimated glomerular filtration rate; HI—hemorrhagic infarction; INR—international normalized ratio; IVH—intraventricular hemorrhage; IVT—intravenous thrombolysis; LMWH—low molecular weight heparin; NIHSS—National Institutes of Health Stroke Scale; PH—parenchymal hematoma; SAH—subarachnoid hemorrhage; SDH—subdural hemorrhage; UFH—unfractionated heparin.

**Table 2:** Representation of individual types of hemorrhage in the study population (remote = bleeding outside the area of ischemia).

Type of Hemorrhage	Number of Cases
HI1 only	55
HI2 only	16
PH1 only	12
PH2 only	25
remote + HI1	3
remote + HI2	1
remote + PH1	6
remote + PH2	10
remote only	31

HI—hemorrhagic infarction; PH—parenchymal hematoma.

**Table 3:** Baseline variables.

Parameter	Overall	Parenchymal Hematoma	No Intracranial Hemorrhage	*p*
n	124	27	97	
Demographics				
Age; years, median [IQR]	78.0 [71.0, 85.0]	78.0 [73.5, 84.5]	78.0 [71.0, 85.0]	0.705
Female sex; n (%)	53 (42.7)	14 (51.9)	39 (40.2)	0.389
Vascular risk factors				
Arterial hypertension; n (%)	103 (83.1)	20 (74.1)	83 (85.6)	0.263
Diabetes mellitus; n (%)	54 (43.5)	6 (22.2)	48 (49.5)	**0.021**
Pre-stroke pharmacotherapy				
Antihypertensives; n (%)	83 (66.9)	18 (66.7)	65 (67.0)	1.000
Antidiabetics; n (%)	47 (37.9)	5 (18.5)	42 (43.3)	**0.034**
Antiplatelets				
ASA; n (%)	34 (27.4)	9 (33.3)	25 (25.8)	0.593
Clopidogrel; n (%)	12 (9.7)	2 (7.4)	10 (10.3)	0.934
Anticoagulation therapy				
Warfarin; n (%)	4 (3.2)	0 (0.0)	4 (4.1)	0.648
Dabigatran; n (%)	2 (1.6)	0 (0.0)	2 (2.1)	1.000
Rivaroxaban; n (%)	0 (0.0)	0 (0.0)	0 (0.0)	
Apixaban; n (%)	1 (0.8)	0 (0.0)	1 (1.0)	1.000
Edoxaban; n (%)	1 (0.8)	0 (0.0)	1 (1.0)	1.000
UFH; n (%)	0 (0.0)	0 (0.0)	0 (0.0)	
LMWH prophylactic; n (%)	0 (0.0)	0 (0.0)	0 (0.0)	
LMWH therapeutic; n (%)	16 (12.9)	1 (3.7)	15 (15.5)	0.198
Clinical parameters				
Systolic blood pressure; mmHg, median [IQR]	155 [145, 170]	160 [147, 187]	155 [143, 170]	0.335
Diastolic blood pressure; mmHg, median [IQR]	80 [74, 94]	80 [70, 96]	80 [74, 90]	0.752
NIHSS; median [IQR]	8 [5, 11]	8 [5, 11]	8 [5, 11]	0.983
ASPECTS; median [IQR]	10 [10, 10]	10 [10, 10] a	10 [10, 10] b	0.644

ASA—acetylsalicylic acid; ASPECTS—Alberta Stroke Program Early CT Score; IQR—interquartile range; LMWH—low molecular weight heparin; n—number; NIHSS—National Institutes of Health Stroke Scale; UFH—unfractionated heparin. Statistically significant differences are highlighted in bold. ^a^ No ASPECT score was recorded in 4 patients with parenchymal hematoma. ^b^ No ASPECT score was recorded in 24 patients without hemorrhagic complications.

**Table 4:** Fibrinogen levels (baseline and after 6 and 24 h), Δ fibrinogen, and fibrinogen ratio.

Parameter	Parenchymal Hematoma	No Intracranial Hemorrhage	*p*
n	20	73	
Fibrinogen baseline; g/L, median [IQR]	3.54 [3.04, 4.90]	3.75 [3.04, 4.27]	0.650
n	27	97	
Fibrinogen 6 h after IVT; g/L, median [IQR]	1.93 [1.31, 2.60]	2.57 [1.95, 3.19]	**0.012**
n	20	75	
Fibrinogen 24 h after; g/L, median [IQR]	2.45 [1.90, 3.28]	2.71 [2.11, 3.34]	0.226
n	20	73	
Δ fibrinogen (baseline vs. 6 h after IVT); g/L, median [IQR]	1.13 [0.91, 2.96]	0.74 [0.35, 2.15]	**0.017**
Fibrinogen ratio (baseline vs. 6 h after IVT); median [IQR]	1.78 [1.37, 3.05]	1.26 [1.11, 2.08]	**0.008**

IQR—interquartile range; IVT—intravenous thrombolysis; n—number. Statistically significant differences are highlighted in bold.

**Table 5:** Dependence of the odds of parenchymal hematoma on fibrinogen levels 6 h after intravenous thrombolysis.

Model	β	$e^{\beta }$	CI	*p*
Parenchymal hematoma				
Fibrinogen level 6 h after IVT [g/L]	−0.55	0.58	0.36–0.88	**0.015**

CI—confidence interval, IVT—intravenous thrombolysis. Statistically significant difference is highlighted in bold.

**Table 6:** Dependence of the odds of parenchymal hematoma on Δ fibrinogen (baseline vs. 6 h after intravenous thrombolysis).

Model	β	$e^{\beta }$	CI	*p*
Parenchymal hematoma				
Δ fibrinogen (baseline vs. 6 h after IVT) [g/L]	0.34	1.41	0.99–2.01	0.057

CI—confidence interval, IVT—intravenous thrombolysis.

**Table 7:** Dependence of the odds of parenchymal hematoma on fibrinogen ratio (baseline vs. 6 h after intravenous thrombolysis).

Model	β	$e^{\beta }$	CI	*p*
Parenchymal hematoma				
Ln (ratio fibrinogen—baseline vs. 6 h after IVT)	0.78	2.18	1.08–4.54	**0.031**

CI—confidence interval, IVT—intravenous thrombolysis; Ln—natural log. Statistically significant difference is highlighted in bold.

**Table 8:** Results of other laboratory parameters.

Parameter	Overall	Parenchymal Hematoma	No Intracranial Hemorrhage	*p*
n	124	27	97	
Urea; mmol/L, median [IQR]	6.24 [5.00, 8.55]	6.31 [5.54, 8.85]	6.20 [4.90, 8.20]	0.327
Creatinine; μmol/L, median [IQR]	87.0 [75.0, 115.3]	92.0 [77.4, 120.5]	85.0 [74.9, 113.0]	0.193
eGFR; mL/s, median [IQR]	1.06 [0.82, 1.31]	0.90 [0.67, 1.14] ^a^	1.10 [0.83, 1.35] ^b^	**0.046**
Platelets before IVT; 10^9^/L, median [IQR]	217.0 [183.5, 262.3]	227.0 [177.5, 271.5]	215.0 [185.0, 256.0]	0.692
Platelets after IVT; 10^9^/L, median [IQR]	210.0 [177.0, 249.5]	202.0 [173.5, 244.5]	212.5 ^c^ [177.8, 250.3]	0.658

eGFR—estimated glomerular filtration rate; IQR—interquartile range; IVT—intravenous thrombolysis. Statistically significant difference is highlighted in bold. ^a^ No eGFR value was recorded in 4 patients with parenchymal hematoma. ^b^ No eGFR value was recorded in 13 patients without hemorrhagic complications. ^c^ The number of platelets after IVT was not recorded in one patient without hemorrhagic complications.

## Data Availability

The datasets generated during and/or analyzed during the current study are available from the corresponding author upon reasonable request.
